# Effect of l-carnitine and emulsifier on growth performance, lipid profile, lipogenesis, and oxidative stress of broiler chickens raised at high altitude

**DOI:** 10.1016/j.psj.2025.105157

**Published:** 2025-04-14

**Authors:** Behnam Ahmadipour, Fathollah Farzam Hasani, Hossein Hassanpour, Fariborz Khajali

**Affiliations:** a Department of Animal Sciences, Faculty of Agriculture, Shahrekord University, Shahrekord, Iran; b Department of Basic Sciences, Faculty of Veterinary Medicine, Shahrekord University, Shahrekord, Iran; c Department of Health Equity Research Center, Shahed University, Tehran, Iran

**Keywords:** L-carnitine, Emulsifier, Lipid profile, Broiler chickens, High altitude

## Abstract

High-altitude environments impose hypobaric hypoxia on broiler chickens, leading to pulmonary hypertension syndrome (**PHS**), oxidative stress, and impaired growth performance. l-carnitine, known for its role in energy metabolism and antioxidant properties, and emulsifiers, which enhance fat digestion, have the potential to mitigate these challenges. This study investigated the effects of l-carnitine and emulsifier supplementation, individually and in combination, on growth performance, lipid metabolism, oxidative stress, and gene expression in broilers raised at high altitudes. A total of 675 male broilers (Ross 308) were randomly assigned to nine dietary treatments, including control, l-carnitine (50 or 100 mg/kg), emulsifier (1 or 2 g/kg), and their combinations. Growth performance, carcass traits, serum lipid profiles, hepatic enzyme activities, oxidative stress markers, and gene expression (CPT1, CPT2, SOD1, iNOS) were evaluated over 42 days. Supplementation with l-carnitine and emulsifier improved weight gain and feed conversion ratio, and reduced feed intake. Carcass yield increased, and abdominal fat decreased significantly in treated groups. l-carnitine reduced serum cholesterol, triglycerides, and LDL while increasing HDL, indicating improved lipid metabolism. Oxidative stress markers, such as malondialdehyde (**MDA**), were reduced, and antioxidant gene expression (SOD1) was upregulated in l-carnitine-supplemented groups. Emulsifiers enhanced fat digestion, as evidenced by increased lipase activity and CPT1/CPT2 gene expression. Combined treatments showed no consistent additive effects. It is concluded that dietary supplementation with l-carnitine and emulsifiers enhances growth performance and lipid metabolism in broilers raised at high altitudes. While l-carnitine enhances antioxidant capacity, reduces oxidative stress, and improves cardiopulmonary health, emulsifiers do not directly affect oxidant and antioxidant status but optimize lipid parameters.

## Introduction

High-altitude environments pose distinct physiological challenges for broiler chickens, primarily due to hypobaric hypoxia (Bhagat et al., 2023). Chickens raised at high altitudes are particularly susceptible to developing pulmonary hypertension syndrome (**PHS**) due to the stress induced by reduced oxygen availability (Hossain and Akter, 2022). The hypoxic conditions lead to the constriction of pulmonary blood vessels, which in turn increases pulmonary vascular resistance and pressure within the lungs. Over time, these adaptations can result in structural changes in the heart, notably right ventricular hypertrophy. The interplay of these factors can culminate in serious health issues, including congestive heart failure and ascites (Khajali, 2022). Additionally, hypoxia can disrupt mitochondrial function, resulting in an increased production of reactive oxygen species (**ROS**). The accumulation of ROS may overwhelm the antioxidant defenses in chickens especially enzymatic antioxidants (superoxide dismutase, **SOD**; catalase, **CAT**; glutathione peroxidase, **GPX**), leading to oxidative stress. This oxidative stress is associated with inflammation, vascular damage, and apoptosis in pulmonary tissues, further aggravating the incidence of PHS (Gaur et al., 2021).

L-carnitine is an important nutrient in poultry nutrition, particularly for broiler chickens. It aids in energy metabolism, enhancing growth performance and improving feed efficiency (Ghoreyshi et al., 2019). Additionally, l-carnitine can support immune function and reduce oxidative stress in chickens, contributing to overall health and productivity. Supplementing l-carnitine in chicken diets is beneficial during periods of stress, such as heat stress or disease challenges (Ghasemi and Nari, 2023). l-carnitine plays a crucial role in the transport of long-chain fatty acids into the mitochondria for energy production. It interacts with enzymes such as carnitine palmitoyltransferases 1 & 2 (**CPT**). CPT1, located on the outer mitochondrial membrane, and CPT2, located in the inner mitochondrial membrane, facilitate the conversion of acyl-CoA to acylcarnitine, allowing fatty acids to cross the mitochondrial membrane. This process is essential for beta-oxidation, where fatty acids are broken down to produce energy (McCann et al., 2021). It has been shown that l-carnitine supplementation can significantly affect lipid profiles and abdominal fat content in chickens (Golrokh et al., 2016).

Emulsifiers are valuable additives in poultry nutrition, contributing to improved chicken performance and nutrient utilization. Recent research has focused on exploring new directions in the use of exogenous and natural emulsifying agents, as well as their blends in poultry diets. These additives are especially effective in enhancing fat digestion and utilization. Various types of emulsifiers are used in poultry diets, including soy-lecithin, milk-derived casein, lysophatidylcholine (lysolecithin), bile salt, glycerol polyethylene glycol ricinoleate (E 484), and sodium stearoyl-2-lactylate (**SSL**) (Sizova and Ryazantseva, 2022). In broilers, the addition of emulsifiers to diets containing poultry fat has been shown to improve digestibility and increase pancreatic secretion (Oketch et al., 2023).

While the individual effects of l-carnitine and emulsifiers on broiler performance and health have been well-documented, their combined use has not been extensively studied in high-altitude environments where hypobaric hypoxia exacerbates metabolic and oxidative stress. The synergistic potential of these additives lies in their complementary mechanisms: l-carnitine enhances intracellular lipid metabolism and antioxidant capacity, while emulsifiers improve fat digestion and absorption. Recent study explored the combined effects of l-carnitine and emulsifiers under normal conditions (Shahmoradi et al., 2022). The combined supplementation of l-carnitine and emulsifiers in a low-energy diet has been shown to yield comparable growth performance to a control diet, while enhancing carcass quality, fat, protein, and dry matter digestibility, as well as blood lipid parameters in broiler chickens. These findings support the rationale for investigating the combined effects of l-carnitine and emulsifiers in high-altitude environments, where hypobaric hypoxia similarly disrupts metabolic and physiological processes. Investigating their combined effects could provide insights into whether they offer additive or synergistic benefits in mitigating the challenges posed by high-altitude rearing.

This study aims to investigate the synergistic effects of l-carnitine and emulsifier supplementation in broiler chickens exposed to high-altitude hypobaric hypoxia, focusing on growth, lipid metabolism, and oxidative stress pathways. The research evaluates different dosage combinations to identify optimal levels for improving production efficiency under environmental stress conditions. Specifically, it assesses the effects on growth performance, serum lipid profiles, hepatic enzyme activities, and lipid peroxidation, as well as the expression of related genes such as CPT1, CPT2, SOD1, and inducible nitric oxide synthase (**iNOS**). By addressing the adverse physiological consequences of hypobaric hypoxia, the study seeks to enhance overall poultry health and productivity, ultimately providing insights into optimizing supplementation strategies for improved outcomes in challenging environmental conditions.

## Materials and methods

### Bird Management and experimental facility

The experiment was conducted at the experimental facility of Shahrekord University, located at an altitude of 2100 meters above sea level in Shahrekord, Iran. The study adhered strictly to the guidelines established by the Guide for the Care and Use Committee of Shahrekord University. A total of 675 day-old male broilers (Ross 308) were randomly allocated to 45-floor pens, each measuring 1.5 m² and housing 15 birds per pen (5 pens per treatment). The chicks had an average weight of 38.5 g, ensuring that all pens started with equal initial body weights of 577.5 ± 10 g. Each pen was equipped with a bell drinker and a feed trough. The chicks were raised on a commercial broiler diet until they reached 5 days of age. Following an eight-hour fasting period and the removal of runts, the five-day-old chicks were allocated to the pens to ensure that each pen had an equal average body weight. Birds were maintained at environmental decreasing temperatures (1-7 days, 32±1°C; 8-14 days, 25±1°C; 15-21 days, 20±1°C; 22-42 days, 15±1°C) (Ahmadipour et al., 2018). Throughout the trial, all chicks had unrestricted access to feed and water and were subjected to a lighting regimen of 23 h of light and 1 h of darkness.

The control diet, formulated based on corn and soybean meal, was designed for three distinct growth phases: starting (1–10 days of age), growing (11–22 days of age), and finishing (23–42 days of age), based on the nutritional requirements of Aviagen (2022) (Table 1). Additionally, eight experimental diets were prepared by incorporating various levels of l-carnitine (Carniking®, Lohmann Co. Ltd., Cuxhaven, Germany) and emulsifier (Lysoforte®, Kemin Co. Ltd., Des Moines, IA) into the control diet. These included formulations with 50 mg/kg l-carnitine (0.005 %), 100 mg/kg l-carnitine (0.01 %), 1 g/kg emulsifier (0.1 %), 2 g/kg emulsifier (0.2 %), 50 mg/kg l-carnitine combined with 1 g/kg emulsifier, 50 mg/kg l-carnitine combined with 2 g/kg emulsifier, 100 mg/kg l-carnitine combined with 1 g/kg emulsifier, and 100 mg/kg l-carnitine combined with 2 g/kg emulsifier.

**Table 1:** Composition of the basal diet fed to broilers (Ross 308) from 1 to 42 days of age.

Item (% unless noted)	Starter (1–10d)	Grower (11-22d)	Finisher (23–42d)
Corn	53.66	56.66	62.38
Soybean meal (44 % CP)	36.95	36.62	30.42
Corn Gluten Meal	3.51	-	-
Soy oil	1.34	2.67	3.47
Dicalcium phosphate	2.15	1.91	1.65
CaCO3	0.68	0.61	0.57
Salt	0.16	0.20	0.19
Na-Bicarbonate	0.29	0.24	0.25
DL-Methionine	0.31	0.29	0.27
L-Lysine	0.28	0.17	0.18
L-Threonine	0.11	0.09	0.07
Choline Chloride	0.06	0.04	0.05
Mineral supplement*	0.25	0.25	0.25
Vitamin supplement⁎⁎	0.25	0.25	0.25
Calculated composition			
Dry Matter	89.026	88.934	88.920
AME (kcal/kg)	2880.00	2950.00	3070.0
CP	22.73	20.579	18.257
Met	0.631	0.574	0.528
Met+Cys	0.910	0.833	0.764
Lys	1.23	1.110	0.980
Thr	0.824	0.744	0.657
Arg	1.31	1.252	1.087
Na	0.157	0.157	0.157
Cl	0.204	0.204	0.204
K	0.931	0.926	0.817
Na+*K*–Cl (mEq/kg)	249.10	247.82	219.92

⁎ Provided the following per kg of diet: vitamin A (trans retinyl acetate), 3600IU; vitamin D3 (cholecalciferol), 800 IU; vitamin E (dl-α-tocopheryl acetate), 7.2 mg; vitamin K3, 1.6 mg; thiamine, 0.72 mg; riboflavin, 3.3 mg; niacin, 0.4 mg; pyridoxin, 1.2 mg; cobalamine, 0.6 mg; folicacid, 0.5 mg; choline chloride, 200 mg.

⁎⁎ Provided the following per kg of diet: Mn (from MnSO4-H2O), 40 mg; Zn (from ZnO), 40 mg; Fe (from FeSO4-7H2O), 20 mg; Cu (from CuSO4-5H2O), 4 mg; I [from Ca (IO3)2-H2O], 0.64 mg; Se (from sodium selenite),0.08 mg.

### Carcass and biochemical measurements

Body weight gain, feed intake, and feed conversion rate were calculated for the following periods: 1 to 10 days, 11 to 22 days, 23 to 42 days, and the overall period from 1 to 42 days. At 42 days of age, 10 birds per treatment group were randomly selected for blood collection and subsequent processing. Blood samples (4 mL) were obtained from the brachial vein and centrifuged at 2500 g for 10 min to separate the serum. The serum samples were then analyzed for various biochemical parameters, including lipase, cholesterol, triglycerides, high-density lipoprotein (**HDL**), low-density lipoprotein (**LDL**), alanine transaminase (**ALT**), aspartate transaminase (**AST**), alkaline phosphatase (**ALP**), and malondialdehyde (**MDA**). As a previous study determined, serum lipase levels can serve as evidence of pancreatic secretion activity and digestive efficiency in broiler chickens (Vertiprakhov et al., 2018). The concentration of serum MDA, a biomarker of oxidative stress, was determined using the method described previously (Hassanpour et al., 2015). Serum lipid profiles and hepatic enzyme activities were measured using standard kits from Pars Azmun Co. (Karaj, Iran). Additionally, blood samples were collected in microhematocrit tubes for hematocrit measurement. Following blood collection, the birds were euthanized, and data collected during processing included live body weight, hot carcass weight, breast weight, and thigh weight. The heart, liver, spleen, bursa, and abdominal fat were also excised and weighed. The heart ventricles were dissected and weighed to calculate the right-to-total ventricular weight ratio (**RV:TV** ratio), which serves as an indicator of PHS. Furthermore, mortality due to PHS was monitored daily throughout the trial, and instances where the RV:TV ratio exceeded 0.25 were considered indicative of pulmonary hypertension (Sharifi et al., 2016).

### RNA extraction, cDNA synthesis, and quantitative real-time PCR analysis

The heart (right ventricle), liver, and lung tissues from 10 euthanized broilers were collected, quickly frozen in liquid nitrogen, and stored at −70°C for RNA analysis. Total RNA extraction was performed using the RNXPlus reagent (Sinaclon Bioscience, Tehran, Iran). A 100 mg homogenized tissue sample was prepared in a digestion buffer and mixed with chloroform. After centrifugation, the total RNA was isolated from the upper aqueous phase. The RNA was precipitated with isopropanol and washed with 75 % ethanol before being resuspended in DEPC-treated water. To eliminate any residual DNA, the RNA underwent DNase treatment (Sinaclon Bioscience, Tehran, Iran) and was quantified using spectrophotometry. Only RNA samples with an absorbance ratio (A260/A280) greater than 1.9 were selected for cDNA synthesis.

The total RNA was reverse transcribed into cDNA utilizing a First Strand cDNA Synthesis Kit (Pars Toos, Mashhad, Iran). The reverse transcription mix was subjected to a brief heating at 85°C for 5 s to inactivate the reverse transcriptase and denature the RNA, followed by storage at −20°C.

Gene expression levels of SOD1, iNOS, CPT1, CPT2, and tyrosine 3-monooxygenase/tryptophan 5-monooxygenase activation protein zeta (**YWHAZ)** were quantified through real-time PCR with RealQ Plus 2x Master Mix Green (Ampliqon, Odense, Denmark). YWHAZ served as the endogenous control for normalizing cDNA input across samples (Hassanpour et al., 2018). Specific primers for SOD1, iNOS, CPT1, CPT2, and YWHAZ were designed according to Abaszadeh et al. (2023), with primer details in Table 2. PCR reactions were conducted in a real-time PCR cycler (Rotor-Gene Q 6000, Qiagen, Hilden, Germany) in triplicate for each ventricular sample. Each reaction consisted of 0.5 µl cDNA combined with 5 µl of Master Mix Green and 0.5 mM of each specific primer in a total volume of 10 µl. The thermal cycling conditions included an initial denaturation at 95°C for 10 min followed by 40 cycles of denaturation at 94°C for 15 s and annealing/extension at temperatures ranging from 60 to 63°C for 10 to 30 s. Fluorescence measurements were taken at the end of each cycle for quantitative analysis.

**Table 2:** Details of the primers used for quantitative real time PCR analysis for chickens.

Target	Primers	PCR product (bp)	Accession no.
YWHAZ	5′-AGGAGCCGAGCTGTCCAATG-3′ 5′-CTCCAAGATGACCTACGGGCTC-3′	84	NM_001031343.1
SOD1	5′-CACTGCATCATTGGCCGTACCA-3′ 5′-GCTTGCACACGGAAGAGCAAGT-3′	224	NM_205064.1
iNOS	5′-AGGCCAAACATCCTGGAGGTC-3′ 5′-TCATAGAGACGCTGCTGCCAG-3′	371	U46504
CPT1	5′-TGTGAGTGATTGGTGGGAAGAG-3′- 5′-GCTGCCTGTATGGTTGTGGG-3′	117	NM_001012898.1
CPT2	5′-GGGTCGTGTTGGGCTGTT-3′ 5′-CTGGGCAGGCTCTTCTGGTA-3′	106	NM_001031287.3

CPT, Carnitine palmitoyl transferase; YWHAZ, tyrosine 3-monooxygenase/tryptophan 5-monooxygenase activation protein zeta; SOD1, superoxide dismutase 1; iNOS, inducible nitric oxide synthase; bp, base pair.

Gene expression data were normalized to YWHAZ levels. LinRegPCR software version 2012.0 (Amsterdam, Netherlands) was used to analyze the data to determine threshold cycle numbers and reaction efficiencies. The relative transcript levels and fold changes in transcript abundance were calculated using the efficiency-adjusted Paffl methodology (Dorak, 2007).

### Statistical analysis

Data were analyzed using the General Linear Model (**GLM**) in SAS software (2007) within a completely randomized design. For data collected from pens with multiple samples, a nested design was not applied because the experimental design did not involve hierarchical or nested factors. Instead, the treatments were applied directly to the pens, and the data were analyzed at the pen level. The statistical model for growth performance data was expressed as: Y_ij_=µ+T_i_+e_ij_. For other variables, the model was formulated as Y_ijk_=µ+T_i_+e_ij_+ɛ_ijk_​. In these equations, Yij and Yijk represent the observations; μ denotes the overall mean; Ti indicates the treatment effect; eij accounts for random error; and ɛ_ijk_ represents subsampling error. Means were differentiated using Duncan's multiple-range test. Interactions between treatments were evaluated within the GLM framework, and significant interactions were reported in the results where applicable.

## Results

### Growth performance indices

Table 3 presented the effect of l-carnitine and emulsifier on the performance parameters and mortality rate of broiler chickens raised at high altitudes after 42 days.

**Table 3:** Effect of l-carnitine and emulsifier on the performance parameters and mortality rate of broiler chickens raised at high altitude after 42 days.

Parameters	Control	Carnitine-0.005 %	Carnitine-0.01 %	Emulsifier-0.1 %	Emulsifier-0.2 %	Carnitine-0.005 % + Emulsifier-0.1 %	Carnitine-0.005 % + Emulsifier-0.2 %	Carnitine-0.01 % + Emulsifier-0.1 %	Carnitine-0.01 % + Emulsifier-0.2 %	SEM	p-value
Feed intake (g/bird)											
1-10 d	242.5ᵃ	236.7ᵃᵇ	231.1ᵇᶜ	232.5ᵃᵇᶜ	234.0ᵃᵇᶜ	233.4ᵃᵇᶜ	228.6ᵇᶜ	223.6ᶜ	234.9ᵃᵇ	1.10	0.025
11-22 d	890.2	861.1	890.4	863.3	854.2	888.5	872.8	879.4	868.6	4.04	0.284
23-42 d	2926.8ᵃ	2703.9ᵇ	2735.0ᵇ	2696.2ᵇ	2737.5ᵇ	2745.9ᵇ	2698.3ᵇ	2603.9ᵇ	2708.3ᵇ	18.20	0.028
1-42 d	4059.4ᵃ	3801.7ᵇ	3855.5ᵇ	3792.0ᵇ	3825.7ᵇ	3867.9ᵇ	3799.7ᵇ	3706.9ᵇ	3811.7ᵇ	19.89	0.022
Weight gain (g/bird)											
1-10 d	190.4	191.3	192.1	191.9	195.8	191.8	190.2	193.3	197.5	1.07	0.776
11-22 d	557.4^b^	579.1^ab^	601.3^a^	586.7^a^	572.2^ab^	592.1^a^	590.6^a^	600.0^a^	585.9^a^	2.98	0.031
23-42 d	1385.3	1409.8	1436.3	1415.2	1459.8	1461.0	1435.4	1394.7	1448.9	10.02	0.559
1-42 d	2113.2ᵇ	2180.3^a^	2229.7^a^	2193.8^a^	2227.8^a^	2224.8^a^	2216.1^a^	2188.1^a^	2232.3^a^	7.64	0.015
Feed conversion ratio (g:g)											
1-10 d	1.29^a^	1.23^b^	1.22^b^	1.21^b^	1.19^bc^	1.21^b^	1.20^b^	1.15^c^	1.19^bc^	0.004	> 0.001
11-22 d	1.59^a^	1.48^b^	1.48^b^	1.47^b^	1.49^b^	1.50^b^	1.48^b^	1.46^b^	1.48^b^	0.006	0.001
23-42 d	2.11^a^	1.91^b^	1.90^b^	1.91^b^	1.88^b^	1.87^b^	1.88^b^	1.86^b^	1.87^b^	0.006	> 0.001
1-42 d	1.90^a^	1.74^b^	1.72^bc^	1.73^bc^	1.71^bc^	1.72^bc^	1.71^bc^	1.69^c^	1.70^bc^	0.004	> 0.001
Mortality rate											
%	8.00	4.00	2.00	6.00	6.00	6.00	2.00	6.00	4.00	1.05	0.530

SEM, standard error of mean; a,b,csignificant differences between groups in each raw.

Feed intake was reduced in the l-carnitine-0.01 % group, l-carnitine-0.005 % + emulsifier-0.2 % group, and l-carnitine-0.01 % + emulsifier-0.1 % group compared to the control group during the 1-10 days (*P* < 0.05). Feed intake also decreased in all treatment groups compared to the control group during the 23-42 and 1-42 days (*P* < 0.05). However, no differences in feed intake were observed between groups during the 11-22 days (*P* > 0.05).

The weight gain increased in all treatment groups compared to the control group during 11-22 (except for l-carnitine-0.005 % and emulsifier-0.2 %) and 1-42 days of rearing (*P* < 0.05) while it did not change between groups during 1-10 and 23-42 days (*P* > 0.05).

The feed conversion ratio decreased in all treatment groups compared to the control group across all rearing periods (*P* < 0.05).

The mortality rate did not change across all experimental groups (*P* > 0.05).

### Carcass parameters

Table 4 compares the effect of l-carnitine and emulsifier on the carcass indices of broiler chickens raised at high altitudes after 42 days.

**Table 4:** Effect of l-carnitine and emulsifier on the carcass indices of broiler chickens raised at high altitude after 42 days.

BW(%)	Control	Carnitine-0.005 %	Carnitine-0.01 %	Emulsifier-0.1 %	Emulsifier-0.2 %	Carnitine-0.005 % + Emulsifier-0.1 %	Carnitine-0.005 % + Emulsifier-0.2 %	Carnitine-0.01 % + Emulsifier-0.1 %	Carnitine-0.01 % + Emulsifier-0.2 %	SEM	p-value
Carcass yield	75.20^c^	76.38^ab^	76.74^a^	75.83^b^	85.85^b^	75.80^b^	75.98^b^	76.00^b^	76.04^b^	0.062	> 0.001
Breast yield	29.12	30.00	29.75	29.38	29.93	28.10	29.74	30.43	29.50	0.175	0.642
Thigh yield	26.33^b^	27.35^a^	27.43^a^	27.29^a^	27.39^a^	27.48^a^	27.43^a^	27.39^a^	27.88^a^	0.095	0.04
Liver	2.27^a^	2.00^bc^	2.13^ab^	1.91^bc^	2.03^bc^	1.98^bc^	2.07^bc^	1.93^bc^	1.99^bc^	0.020	0.006
Spleen	0.08^c^	0.11^ab^	0.13^a^	0.10^bc^	0.10^bc^	0.10^bc^	0.10^bc^	0.11^ab^	0.12^ab^	0.003	0.007
Bursa	0.07^c^	0.11^a^	0.12^a^	0.09^b^	0.11^ab^	0.11^ab^	0.12^a^	0.11^a^	0.12^a^	0.002	> 0.001
Abdominal fat	1.10^a^	0.66^b^	0.61^b^	0.49^b^	0.57^b^	0.54^b^	0.58^b^	0.60^b^	0.48^b^	0.023	> 0.001
heart	0.61^a^	0.52^bc^	0.50^c^	0.53^bc^	0.57^ab^	0.51^c^	0.50^c^	0.50^c^	0.52^bc^	0.006	0.003
RV	0.13^a^	0.09^b^	0.08^b^	0.11^ab^	0.11^ab^	0.09^b^	0.09^b^	0.09^b^	0.12^ab^	0.003	0.03
TV	0.44	0.36	0.41	0.42	0.42	0.37	0.39	0.38	0.42	0.011	0.756
RV:TV	0.30^a^	0.24^bc^	0.20^c^	0.26^ab^	0.26^ab^	0.24^bc^	0.23^bc^	0.25^bc^	0.26^ab^	0.005	0.005

BW, body weight; RV, right ventricle; TV, total ventricle; SEM, standard error of mean; a,b,csignificant differences between groups in each raw.

The emulsifier-0.2 % group demonstrated the highest carcass yield, significantly higher than the control group and all other treatment groups (*P* < 0.05). The carcass yield in the l-carnitine-0.01 % group was greater than the control and all combinations of l-carnitine and emulsifier groups (*P* < 0.05). The comparison of carcass yield between other groups was not significant (*P* > 0.05).

The thigh yield was higher in all treatment groups than in the control group (*P* < 0.05), with no differences between treatments (*P* > 0.05).

The control group exhibited the highest liver yield, which was greater than all other treatment groups except for l-carnitine-0.01 % (*P* < 0.05). Treatment groups did not differ for liver yield (*P* > 0.05).

The spleen yield in the l-carnitine-0.01 % group was higher than other experimental groups, except for two groups of l-carnitine-0.01 % + emulsifier-0.1 & 0.2 %. In contrast, the control group had a lower spleen yield than other groups except for two emulsifier groups and two l-carnitine-0.005 % + emulsifier-0.1 & 0.2 % groups (*P* < 0.05).

The control group showed the lowest value of bursa yield, while the groups containing carnitine and emulsifier demonstrated higher values than other groups (*P* < 0.05).

The control group exhibited the highest abdominal fat percentage while all treatment groups showed lower values (*P* < 0.05). Treatment groups did not differ for this parameter.

The heart weight percentage was higher in the control group than in the other experimental groups, except for the emulsifier-0.2 % group (*P* < 0.05). In contrast, no differences are observed among the other groups (*P* > 0.05).

RV% and RV:TV% were reduced in l-carnitine groups (0.005 % and 0.01 %) and l-carnitine + emulsifier groups (except for l-carnitine-0.01 % + emulsifier-0.2 %) compared to the control group (*P* < 0.05). No differences were found between the control and two emulsifier groups (*P* > 0.05).

Breast yields and TV% showed no differences across the experimental groups (*P* > 0.05).

### Biochemical parameters

Table 5 compares the effect of l-carnitine and emulsifier on the biochemical parameters of broiler chickens raised at high altitudes after 42 days.

**Table 5:** Effect of l-carnitine and emulsifier on the biochemical parameters of broiler chickens raised at high altitude after 42 days.

**Metabolites**	Control	Carnitine-0.005 %	Carnitine-0.01 %	Emulsifier-0.1 %	Emulsifier-0.2 %	Carnitine-0.005 % + Emulsifier-0.1 %	Carnitine-0.005 % + Emulsifier-0.2 %	Carnitine-0.01 % + Emulsifier-0.1 %	Carnitine-0.01 % + Emulsifier-0.2 %	SEM	p-value
Cholesterol (mg/dL)	160.0	137.4ᵇᶜ	122.4ᶜ	146.0ᵃᵇ	153.8ᵃᵇ	149.4ᵃᵇ	145.0ᵃᵇ	143.0ᵃᵇ	149.6ᵃᵇ	1.949	0.005
Triglycerides (mg/dL)	102.2ᵃ	77.4ᶜ	75.0ᶜ	93.0ᵃᵇ	95.2ᵃᵇ	84.2ᵇᶜ	85.0ᵇᶜ	83.6ᵇᶜ	81.6ᵇᶜ	1.432	0.001
HDL (mg/dL)	64.6ᶜ	78.9ᵃᵇ	88.2ᵃ	75.3ᵇᶜ	74.9ᵇᶜ	82.3ᵃᵇ	78.3ᵃᵇ	75.5ᵃᵇ	85.0ᵃᵇ	1.264	0.003
LDL (mg/dL)	37.4ᵃ	29.7ᶜ	28.8ᶜ	32.9ᵇᶜ	34.7ᵃᵇ	31.4ᵇᶜ	30.9ᵇᶜ	30.2ᵇᶜ	30.2ᵇᶜ	0.492	0.003
Lipase (U/L)	0.013ᶜ	0.015ᵃᵇᶜ	0.014ᵇᶜ	0.021ᵃ	0.022ᵃ	0.020ᵃᵇ	0.020ᵃᵇ	0.021ᵃ	0.021ᵃ	0.001	0.015
Malondialdehyde (µmol/L)	3.59ᵃ	2.67ᵇ	2.05ᶜ	3.08ᵃᵇ	3.21ᵃᵇ	2.79ᵇ	2.67ᵇ	2.87ᵇ	2.95ᵇ	0.065	> 0.001
Hematocrit (%)	49.0ᵃ	39.2ᵇ	37.6ᵇ	42.0ᵇ	41.4ᵇ	39.0ᵇ	41.6ᵇ	37.8ᵇ	40.4ᵇ	0.668	0.003
ALT (IU/L)	5.6ᵃ	3.2ᶜ	3.0ᶜ	3.8ᵇᶜ	4.4ᵇ	3.4ᶜ	3.4ᶜ	3.4ᶜ	3.4ᶜ	0.097	> 0.001
AST (U/L)	382.8ᵃ	250.8ᵈᵉ	228.0ᵉ	323.4ᵃᵇᶜ	334.2ᵃᵇ	279.6ᵇᶜᵈᵉ	299.8ᵇᶜᵈ	274.4ᵇᶜᵈᵉ	259.8ᶜᵈᵉ	7.007	> 0.001
ALP (U/L)	1012.8ᵃ	471.8ᵇᶜ	385.8ᶜ	844.6ᵃ	973.2ᵃ	588.4ᵇ	580.2ᵇᶜ	569.6ᵇᶜ	555.2ᵇᶜ	21.09	> 0.001

SEM, standard error of the mean; a,b,c significant differences between groups in each raw (*P* < 0.05).

Cholesterol levels were decreased in two l-carnitine (0.005 and 0.01 %) groups compared to the control group (*P* < 0.05) while there were no differences between other groups (*P* < 0.05).

Triglyceride levels were reduced in two l-carnitine (0.005 % and 0.01 %) groups and combined with emulsifiers compared to the control group (*P* < 0.05). The two emulsifier (0.1 % and 0.2 %) groups showed no difference compared to the control group (*P* > 0.05). Differences between other groups were also not significant.

The l-carnitine-0.01 % group exhibited the highest HDL levels compared to the other groups. All other treatment groups also had higher HDL levels than control groups (*P* < 0.05), except for the two emulsifier groups (0.1 % and 0.2 %), which did not show this increase.

All treatment groups showed lower LDL levels than the control group (*P* < 0.05) except for the emulsifier-0.2 % group.

The lipase levels increased in all treatment groups compared to the control group (*P* < 0.05) except for two l-carnitine (0.005 % and 0.01 %) groups. There were no differences between treatment groups (*P* > 0.05).

The MDA levels were lower in all treatment groups than the control group (*P* < 0.05) except for two emulsifier (0.1 % and 0.2 %) groups which did not show this decrease.

The hematocrit was lower in all treatment groups than in the control group (*P* < 0.05). Differences between treatment groups were not significant.

ALT levels decreased in all treatment groups compared to the control group (*P* < 0.05). The AST and AST levels were also reduced in all treatment groups compared to the control group (*P* < 0.05) except for the two emulsifier groups (0.1 % and 0.2 %).

### Gene expression of CPT1, CPT2, SOD1, and iNOS

Table 6 compares the effect of l-carnitine and emulsifier on the gene expression in the liver, heart, and lung of broiler chickens raised at high altitude after 42 days.

**Table 6:** Effect of l-carnitine and emulsifier on gene expression in the liver, heart and lung of broiler chickens raised at high altitude after 42 days.

Gene	Control	Carnitine-0.005 %	Carnitine-0.01 %	Emulsifier-0.1 %	Emulsifier-0.2 %	Carnitine-0.005 % + Emulsifier-0.1 %	Carnitine-0.005 % + Emulsifier-0.2 %	Carnitine-0.01 % + Emulsifier-0.1 %	Carnitine-0.01 % + Emulsifier-0.2 %	SEM	p-value
**liver**											
CPT1	0.002^c^	0.027^bc^	0.015^bc^	0.117^ab^	0.139^a^	0.034^bc^	0.089^abc^	0.064^abc^	0.078^abc^	0.030	0.042
CPT2	0.092^b^	0.108^b^	0.103^b^	0.363^ab^	0.642^a^	0.241^ab^	0.269^ab^	0.469^ab^	0.593^a^	0.124	0.015
iNOS	0.003^b^	0.276^b^	0.230^a^	0.004^b^	0.006^b^	0.061^b^	0.050^b^	0.223^a^	0.217^a^	0.053	0.003
**heart**											
CPT1	0.017^c^	0.062^c^	0.027^c^	0.304^b^	0.510^a^	0.030^c^	0.096^c^	0.088^c^	0.056^c^	0.044	0.001
CPT2	0.015^b^	0.031^b^	0.020^b^	0.392^a^	0.453^a^	0.018^b^	0.284^ab^	0.247^ab^	0.286^ab^	0.098	0.023
SOD1	0.002^c^	0.034^bc^	0.223^a^	0.002^c^	0.005^c^	0.055^bc^	0.049^bc^	0.185^ab^	0.186^ab^	0.048	0.018
**lung**											
CPT1	0.001^b^	0.049^b^	0.026^b^	0.372^ab^	0.709^a^	0.059^b^	0.255^b^	0.118^b^	0.211^b^	0.132	0.029
CPT2	0.004^c^	0.004^c^	0.004^c^	0.413^ab^	0.585^a^	0.086^c^	0.220^bc^	0.074^c^	0.183^bc^	0.091	0.002
SOD1	0.010^c^	0.082^bc^	0.214^a^	0.017^c^	0.048^bc^	0.032^bc^	0.082^bc^	0.132^ab^	0.129^ab^	0.031	0.004

SEM, standard error of the mean; a,b,c significant differences between groups in each raw (*P* < 0.05).

The relative expression of the CPT1 gene was higher in the emulsifier groups (0.1 % and 0.2 %) in both liver and heart tissues compared to the control group (*P* < 0.05). In the lung tissue, an increase of CPT1 gene expression was observed only in the emulsifier-0.2 % group (*P* < 0.05). Other treatment groups did not change compared to the control (*P* > 0.05).

The relative expression of the CPT2 gene was elevated in the emulsifier groups (0.1 % and 0.2 %) in both lung and heart tissues compared to the control group. In the liver, increased expression was observed only in the emulsifier-0.2 % and the Carnitine-0.01 % + emulsifier-0.2 % groups. Other treatment groups did not change compared to the control group (*P* > 0.05).

The relative expression of the SOD1 gene was higher in the l-carnitine-0.01 %, l-carnitine-0.01 % + emulsifier-0.1 %, and l-carnitine-0.01 % + emulsifier-0.2 % groups in both lung and heart tissues compared to the control group. In the lung tissue, an increase of CPT1 gene expression was observed only in the emulsifier-0.2 % group. Other treatment groups did not change compared to the control (*P* > 0.05).

The relative expression of the iNOS gene was higher in the l-carnitine-0.01 %, l-carnitine-0.01 % + emulsifier-0.1 %, and l-carnitine-0.01 % + emulsifier-0.2 % groups in liver tissues compared to the control and other groups. The expression of the iNOS gene did not change across other treatment and control groups (*P* > 0.05).

## Discussion

This study provides comprehensive insights into the effects of l-carnitine and emulsifier supplementation on broiler chickens raised at high altitudes, where hypobaric hypoxia poses significant physiological challenges. The results demonstrate that these dietary additives improve growth performance, lipid metabolism, antioxidant capacity, and cardiopulmonary health, offering a promising strategy to mitigate the adverse effects of high-altitude stress. However, it is important to clarify that this study did not directly investigate fat digestion, and any inferences about fat digestion are based on indirect measures such as lipase activity and gene expression related to lipid metabolism.

The positive changes in feed conversion observed in this study are consistent with the well-known functions of l-carnitine and emulsifiers in improving energy use and nutrient uptake (El-Saway et al., 2022). This process not only provides energy for growth but also reduces the accumulation of lipids in tissues, contributing to lean body composition. The reduced feed intake in supplemented groups during the early rearing phase suggests that these additives enhance feed efficiency, allowing birds to achieve comparable growth with less feed (Azizi-Chekosari et al., 2021). This may be beneficial in high-altitude environments, where hypoxia can impair feed intake and nutrient absorption (Khajali and Wideman, 2016). The average body weight gain in this study was lower than the standard expected for Ross 308 broilers under optimal conditions. This discrepancy can be attributed to factors related to the high-altitude environment and experimental conditions. The hypobaric hypoxia at 2100 meters above sea level likely imposed significant metabolic stress on the birds, reducing their growth potential. Although l-carnitine and emulsifier supplementation improved growth performance compared to the control group, the overall growth rate remained below the standard, suggesting that additional nutritional or management strategies may be needed to fully overcome the limitations of high-altitude rearing.

The findings of improved carcass traits in this study highlight the potential of l-carnitine and emulsifiers to optimize meat quality and productivity in high-altitude poultry production. Organ health also benefited, with lower liver and heart weights, reduced PHS, and enhanced immune organ weights. However, these data are consistent with other studies (Ghoreyshi et al., 2019; Ko et al., 2023).

Sizova and Ryazantseva (2022) determined that emulsifiers improve fat digestion by enhancing micelle formation and increasing the activity of pancreatic lipase. This leads to better absorption of dietary fats, which are a concentrated energy source. The synergistic effects of l-carnitine and emulsifiers were evident in the combined treatment groups, where improvements in FCR were more pronounced. However, the lack of additive effects in some combined treatments suggests that the benefits may plateau at certain doses, highlighting the need for further optimization of supplementation levels.

Although our study did not directly assess fat digestion, the elevated lipase activity in groups receiving emulsifier supplementation implies enhanced fat utilization, which likely contributed to the improved growth performance observed in these birds. This aligns with their role in boosting nutrient utilization, particularly in high-altitude environments where hypoxia impairs digestion (de Oliveira et al., 2019). In contrast, l-carnitine did not increase lipase activity, as it primarily enhances intracellular lipid metabolism (fatty acid oxidation) rather than digestive processes. Combining l-carnitine and emulsifiers showed no additive effect on lipase activity, suggesting emulsifiers drive digestive improvements while l-carnitine optimizes fat utilization post-absorption. These findings highlight their complementary roles: emulsifiers improve fat digestion, and l-carnitine enhances energy production from absorbed fats.

The alterations in serum lipid parameters observed in our study among carnitine-supplemented groups may be evidence of enhanced lipid catabolism and improved lipid profiles. Previous studies reported that l-carnitine reduces the availability of substrates for hepatic lipogenesis, lowering circulating lipid levels (Bahrampour et al., 2024; Eskandani et al., 2022). This is particularly relevant in high-altitude environments, where hypoxia can disrupt lipid metabolism, leading to fat accumulation in tissues and exacerbating oxidative stress and cardiovascular strain. The reduction in abdominal fat percentage in all treatment groups further supports the hypothesis that l-carnitine and emulsifiers promote lean mass accretion by redirecting energy from fat deposition to growth. This is consistent with a previous study showing that l-carnitine supplementation reduces fat deposition in broilers (Corduk et al., 2007). Emulsifiers, by improving fat digestibility, likely contribute to this effect by ensuring that dietary fats are efficiently utilized for energy rather than stored (Oketch et al., 2023).

As mentioned, high-altitude hypoxia overwhelms endogenous antioxidant defenses through ROS production, leading to oxidative stress (Gaur et al., 2021). The reduction in MDA, a marker of lipid peroxidation, in l-carnitine–supplemented groups highlights its antioxidant properties. As demonstrated by Surai (2015), l-carnitine enhances mitochondrial efficiency, reducing electron leakage and ROS production, a mechanism that effectively decreases oxidative stress. The upregulation of SOD1 in cardiac and pulmonary tissues in high-dose l-carnitine groups further supports enhanced antioxidant defense. SOD1 is a critical enzyme that converts superoxide radicals into less harmful molecules, protecting tissues from oxidative damage (Li et al., 2012; Surai, 2016). The lack of significant MDA reduction in emulsifier-only groups suggests that their primary benefits lie in digestion and nutrient absorption rather than direct antioxidant activity.

The RV:TV ratios in l-carnitine and combination groups indicated improved PHS as the major concern in broilers raised at high altitudes. This likely stems from enhanced mitochondrial function, which reduces hypoxia-induced vasoconstriction and improves oxygen utilization. l-carnitine may alleviate the risk of PHS by supporting energy production in cardiac and pulmonary tissues, thereby reducing the workload on the heart and preventing right ventricular hypertrophy. Furthermore, the elevated expression of iNOS in the high-dose l-carnitine groups suggests an additional mechanism through which l-carnitine may mitigate PHS. Nitric oxide (**NO**), produced via iNOS, plays a dual role in vasodilation and immune modulation, which could further improve vascular function and reduce hypertension in broilers under hypoxic stress. It has also been shown that l-carnitine, through the enhancement of NO bioavailability, improves vascular health and reduces the risk of pulmonary hypertension, which supports our findings (Yousefi et al., 2013).

The upregulation of CPT1 and CPT2 in emulsifier-supplemented groups, particularly in hepatic and cardiac tissues, aligns with enhanced fatty acid oxidation, which may improve myocardial energy supply under hypoxic stress. Interestingly, l-carnitine alone did not upregulate CPT genes, suggesting that its effects are post-transcriptional, possibly enhancing enzyme activity rather than expression. This finding contrasts with earlier studies that indicated increased CPT gene expression due to l-carnitine supplementation (Godárová et al., 2005; Shahouzehi et al., 2022). This discrepancy could arise from differences in species, study design, or the specific conditions under which the experiments were conducted. Additionally, variations in the dosage and duration of l-carnitine supplementation may also contribute to differing results.

The study demonstrates that dietary supplementation with l-carnitine and emulsifiers significantly improves growth performance, lipid metabolism, and antioxidant capacity, while also mitigating the adverse effects of hypobaric hypoxia, such as pulmonary hypertension and oxidative damage, in broiler chickens raised at high altitudes. l-carnitine enhances energy metabolism, reduces oxidative stress, and improves lipid profiles, whereas emulsifiers optimize fat digestion and nutrient utilization. However, the combined supplementation did not consistently exhibit additive or synergistic effects. These findings underscore the potential of l-carnitine and emulsifiers as effective dietary strategies to enhance productivity and health in high-altitude poultry farming.

While the l-carnitine content and nutrient profiles of the diets were calculated based on the composition of the raw materials and the known concentration of the supplemented additives, we acknowledge that direct analysis of l-carnitine content and nutrient profiles in all diets would have provided additional validation of the dietary treatments. This is a limitation of our study, and we recommend that future studies include such analyses to further strengthen the reliability of the findings.

## Declaration of competing interest

The authors declare no conflicts of interest.
